# The relationship between the non–high-density lipoprotein cholesterol to high-density lipoprotein cholesterol ratio and tubular atrophy/interstitial fibrosis in patients with IgA nephropathy

**DOI:** 10.1080/0886022X.2026.2699010

**Published:** 2026-07-14

**Authors:** Shuo Li, Yan Huang, Meiran Cao, Jingfu Wang

**Affiliations:** Department of Nephrology, Affiliated Hospital of Chengde Medical University, Chengde, Hebei Province, China

**Keywords:** Non–high-density lipoprotein cholesterol to high-density lipoprotein cholesterol ratio, IgA nephropathy, glomerulonephritis, renal tubule, interstitial fibrosis

## Abstract

**Background: **The tubular atrophy/interstitial fibrosis (T) lesions of the Oxford Classification is a key prognostic determinant in IgA nephropathy (IgAN). The non-high-density lipoprotein cholesterol to high-density lipoprotein cholesterol ratio (NHHR) is an emerging lipid marker, but its link with renal histologic damage in IgAN is unknown.

**Methods: **This cross-sectional and retrospective study investigated the relationship between NHHR and T lesions in 400 biopsy-proven IgAN patients. Participants were divided into NHHR quartiles. The association between NHHR and T lesions was assessed using Spearman’s rank correlation analysis, binary logistic regression analysis, restricted cubic spline (RCS) analysis, receiver operating characteristic (ROC) curve analysis, and subgroup analysis.

**Results: **Compared to the lowest quartile (Q1), the highest NHHR quartile (Q4) showed significantly adverse clinical profiles and a higher prevalence of T1/T2 lesions (*p* < 0.05). NHHR was positively correlated with T lesions (*r* = 0.229, *p* < 0.001). Multivariate logistic regression analysis identified elevated NHHR and reduced estimated glomerular filtration rate (eGFR) as independent risk factors for T1/T2 lesions. RCS analysis demonstrated a linear association between NHHR and the risk of T1/T2 lesions (*p* for nonlinearity = 0.343). The ROC curve analysis yielded an area under the curve (AUC) of 0.635 for NHHR alone (optimal cutoff value = 3.54, sensitivity = 58.2%, specificity = 63.6%), and the composite NHHR+eGFR model achieved an AUC of 0.814. Subgroup analysis showed a consistent association between NHHR and the risk of T1/T2 lesions across all subgroups, with no significant interaction effects.

**Conclusion: **Elevated NHHR is an independent risk factor for the progression of T lesions in patients with IgAN.

## 1. Introduction

IgA nephropathy (IgAN) is a leading cause of primary glomerular disease worldwide. Without clinical intervention, the natural progression of the disease leads to end-stage renal disease in an estimated 20% to 40% of affected individuals within 10 to 20 years [1]. Different degrees of histopathological changes in IgAN contribute to varying disease prognoses. According to research, in the Oxford classification of IgAN, moderate-to-severe renal tubular atrophy/interstitial fibrosis (T1/T2) is an independent predictor of poor renal survival in patients. Irreversible interstitial fibrosis is one of the common final pathways in all chronic and progressive kidney diseases [2]. Currently, percutaneous renal biopsy remains the gold standard for the diagnosis of T lesions. However, its invasive nature and associated procedural risks limit repeated performance, which poses a major challenge in clinical practice. The inability to conduct serial dynamic monitoring of T lesions makes it difficult for clinicians to track lesion status in real time and evaluate treatment responses, thus easily leading to delayed intervention. Therefore, the identification of non-invasive biomarkers for the adjunctive screening of T lesions has become an urgent clinical need. While such low-cost non-invasive indicators cannot replace percutaneous renal biopsy, they can provide a valuable reference for clinicians to early identify IgAN patients with concomitant tubulointerstitial injury.

The detrimental effects of dyslipidemia on kidney health are increasingly recognized. Previous studies have demonstrated that dyslipidemia is associated with the clinical features and renal pathological changes in patients with IgAN and can influence disease progression and prognosis [3]. However, most studies have focused primarily on traditional lipid indices, such as triglycerides (TG) and high-density lipoprotein cholesterol (HDL-C). In recent years, the non–high-density lipoprotein cholesterol to high-density lipoprotein cholesterol ratio (NHHR) has emerged as a novel composite lipid biomarker [4]. This ratio integrates the pro-atherogenic properties of non-HDL-C with the protective characteristics of HDL-C, potentially reflecting the balance between these two lipoprotein classes. Compared with other lipid indices, NHHR has the following advantages: First, NHHR encompasses all atherogenic lipoprotein particles (such as VLDL, IDL, LDL, and Lp(a)), providing a more comprehensive assessment of the overall burden of dyslipidemia. Second, non-HDL-C levels are less affected by short-term fluctuations and fasting status, yielding more stable measurements; therefore, NHHR may serve as a more convenient screening tool [5]^.^ Previous studies have shown that the NHHR has superior predictive value compared with conventional lipid parameters [6,7]. NHHR has been shown to play a significant role in various diseases, including diabetes mellitus, hypertension, depression, osteoporosis, and cholelithiasis [8–12]. Furthermore, several studies have reported a close association between NHHR and kidney diseases. For example, a cohort study involving 3,715 older adults in the United States found that elevated NHHR was significantly associated with an increased risk of chronic kidney disease (CKD) [13]. Pan et al. reported a positive correlation between NHHR and diabetic kidney disease (DKD) [14]. A recent cohort study based on the China Health and Retirement Longitudinal Study (CHARLS) further confirmed that NHHR is a robust predictor of rapid kidney function decline in patients with cardiovascular-kidney-metabolic (CKM) syndrome, demonstrating a significant association with the risk of kidney function deterioration [15].

As a classic immune-mediated glomerular disease, IgAN is characterized by mesangial cell activation as its core pathological feature. This process releases various pro-inflammatory cytokines, which mediate tubulointerstitial fibrosis through pathways such as upregulating TGF-β1 expression and stimulating abnormal matrix protein synthesis by renal tubular epithelial cells [16]. Previous studies have found that patients with low HDL levels have significantly more severe tubulointerstitial fibrosis; LDL and oxidized low-density lipoprotein can directly promote renal tubular epithelial cell transdifferentiation and aggravate fibrosis [17]. These lines of evidence suggest that lipid metabolism is an important target for regulating the inflammation-fibrosis pathway in IgAN. As a novel lipid marker integrating the balance between atherogenic lipoproteins and protective lipoproteins, NHHR can more comprehensively capture the lipid-mediated inflammation and fibrosis signals in IgAN compared with single lipid indicators. Therefore, the association between NHHR and the characteristic T lesions of IgAN is worthy of in-depth investigation.

## 2. Materials and methods

### 2.1. Study population

**Figure 1.**
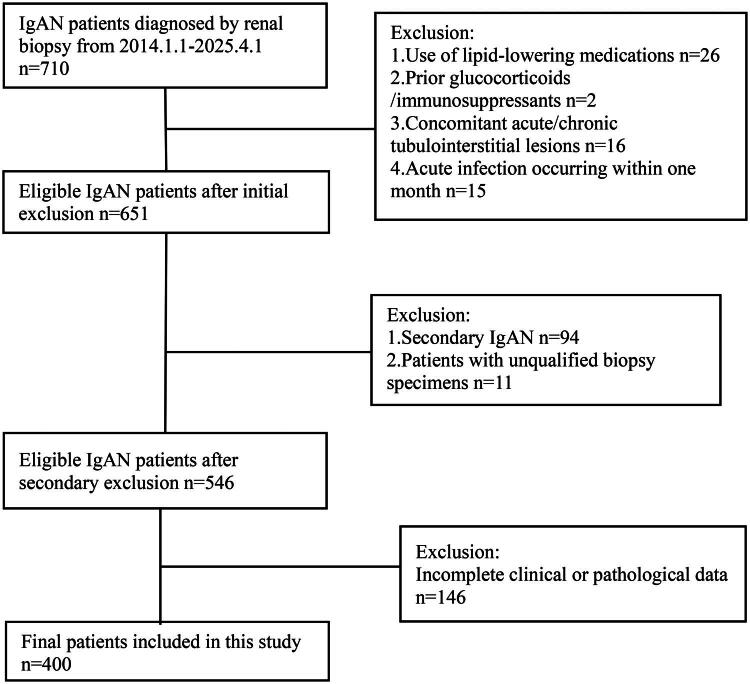
The flow chart of the study.

This study enrolled treatment-naïve patients with primary IgAN who were diagnosed by renal biopsy at the Department of Nephrology, Affiliated Hospital of Chengde Medical University, between January 2014 and April 2025. Clinical and pathological data from renal biopsies were collected. The exclusion criteria were: (1) secondary IgAN, such as that associated with systemic lupus erythematosus, liver cirrhosis, or Henoch-Schönlein purpura;(2) unqualified biopsy specimens;(3) use of lipid-lowering medications;(4) prior use of glucocorticoids or other immunosuppressive agents;(5) concomitant acute or chronic tubulointerstitial lesions; (6) acute infection occurring within one month; and(7) incomplete clinical or pathological data. Ultimately, 400 eligible patients with IgAN were included in the study. The study flow chart is shown in Figure 1. During the data collection phase, completeness checks were performed on all initially screened patients. Cases meeting the exclusion criteria were directly excluded, while those with missing data were removed using complete case analysis, and no form of imputation was performed for missing data.

This study was approved by the Ethics Committee of the Affiliated Hospital of Chengde Medical University (Approval No. CYFYLL2023409). All procedures were performed in accordance with the ethical standards of the Declaration of Helsinki. Written informed consent was obtained from all participants.

### 2.2. Clinical data

Clinical data included general information and laboratory parameters. Demographic information, such as age and sex, was collected from the electronic medical record system. Body weight, height, body mass index (BMI), systolic blood pressure (SBP), diastolic blood pressure (DBP), and history of hypertension were recorded. BMI was calculated as weight (kg)/[height (m)]^2^. For all patients, fasting blood samples were collected on the morning after admission and prior to renal biopsy for biochemical analysis. Laboratory parameters included hemoglobin (Hb), platelet count (PLT), total cholesterol (TC), triglycerides (TG), high-density lipoprotein cholesterol (HDL-C), low-density lipoprotein cholesterol (LDL-C), non–high-density lipoprotein cholesterol (non-HDL-C), albumin (ALB), serum creatinine (Scr), blood urea nitrogen (BUN), uric acid (UA), estimated glomerular filtration rate (eGFR), 24-h urinary protein excretion (24-h urine protein electrophoresis [UPE]), serum immunoglobulin G (IgG), serum immunoglobulin A (IgA), serum complement C3, and serum complement C4.Obesity was defined as a BMI of ≥28 kg/m^2^.Based on the measured lipid levels, NHHR was calculated using the formula: NHHR = (TC – HDL-C)/HDL-C.

### 2.3. Pathological data

All renal tissue specimens were obtained *via* percutaneous renal biopsy. The biopsy specimens were routinely processed for light microscopy, immunofluorescence (IF), and electron microscopy. According to the MEST-C score of the Oxford classification, all renal specimens were assessed and graded based on five key pathological features: M, E, S, T, and C [18]. The criteria were as follows:(1) Mesangial hypercellularity (M): M0, <4 mesangial cells per mesangial area; M1, ≥4 mesangial cells per mesangial area.(2) Endocapillary hypercellularity (E): E0, absent; E1, present.(3) Segmental glomerulosclerosis (S): S0, absent; S1, present.(4) Tubular atrophy/interstitial fibrosis (T): T0, <25% of cortical area involved; T1, 26%–50% involved; T2, >50% involved.(5) Cellular or fibrocellular crescents **(C):** C0, no crescents; C1, crescents in <25% of glomeruli; C2, crescents in ≥25% of glomeruli. The diagnosis of the renal biopsy was initially made independently by two pathologists, and the final diagnosis was determined after a clinicopathological discussion involving all authors. To assess the inter-observer agreement of T lesion grading, 50 renal biopsy sections were randomly selected for re-evaluation. The two aforementioned pathologists, who were double-blinded to the patients’ clinical data and initial pathological diagnosis, performed independent re-grading of T lesions. The overall percent agreement of T lesion grading between the two pathologists was 92.0% (46/50). Further assessment of inter-observer agreement using Cohen’s kappa coefficient yielded a value of 0.854, indicating favorable reliability of the pathological assessment in this study.

### 2.4. Statistical analysis

All statistical analyses were performed using SPSS version 26.0 and R version 3.4.3. Patients were divided into four groups according to the quartiles of NHHR. The normality of continuous variables was assessed using the Shapiro–Wilk test. Normally distributed measurement data are presented as mean ± standard deviation. Normally distributed data were compared among groups using one-way ANOVA with LSD-t post-hoc tests; non-normally distributed data, presented as median (IQR, P25–P75), were compared using the Kruskal-Wallis H test followed by Mann-Whitney U tests with Bonferroni correction for multiple comparisons. Categorical data are expressed as frequencies and percentages, and comparisons between groups were made using the chi-square (χ^2^) test. The correlation between NHHR and T lesions was analyzed using Spearman’s rank correlation analysis. Logistic regression analysis was used to explore the risk factors for the development of T1/T2 lesions. First, univariate logistic regression analysis was performed on all clinical and pathological indicators to preliminarily identify variables associated with T1/T2 lesions. Then, collinearity diagnostics were applied to the screened variables, and those with a variance inflation factor (VIF) >5 were excluded. Finally, multivariable logistic regression analysis was performed to identify independent risk factors for T1/T2 lesions. Variables without significant collinearity and with *p* < 0.05 in univariate analysis were entered into the multivariable model. A potential non-linear relationship between NHHR and T lesions was assessed using restricted cubic splines (RCS). The receiver operating characteristic (ROC) curve was used to evaluate the optimal cut-off value, sensitivity, and specificity of NHHR for predicting T1/T2 lesions. Meanwhile, a calibration curve corrected by 1,000 bootstrap resampling was plotted for the combined model, and the Hosmer-Lemeshow test was used to assess its calibration; furthermore, decision curve analysis (DCA) was performed to evaluate the clinical net benefit of the combined model. Subgroup analysis was conducted to explore the association between NHHR and T lesions across different subgroups, and interaction terms were used to assess the statistical significance of differences between these subgroups. *p* values less than 0.05 were considered statistically significant.

Based on the observed incidence rates of T1/T2 lesions in the lowest and highest NHHR quartiles (24% vs 57%), the required sample size per group to detect this difference with 80% power at a two-sided α of 0.05 was calculated to be 34 patients. The present study included 100 patients per quartile group, indicating adequate statistical power.

## 3. Results

### 3.1. Baseline characteristics of all study subjects

A total of 400 patients were included in this study. Among them, there were 211 males (52.7%) and 189 females (47.3%), with a mean age of 42.24 ± 13.48 years. Based on the quartiles of NHHR, the patients were divided into four groups: Quartile 1 (Q1, <2.61), Quartile 2 (Q2, 2.61–3.39), Quartile 3 (Q3, 3.39–4.14), and Quartile 4 (Q4, >4.14).

There were statistically significant differences among the four groups in sex, body weight, BMI, SBP, DBP, Hb, TG, TC, HDL-C, LDL-C, BUN, Scr, eGFR, UA, C3, C4, and 24-h UPE (*p* < 0.05). Specifically, compared with the Q1 group, the Q4 group had higher levels of body weight, BMI, SBP, DBP, Hb, TG, TC, LDL-C, BUN, Scr, UA, C3, C4, and 24-h UPE, lower levels of HDL-C and eGFR, and a higher proportion of male patients (all *p* < 0.05). No statistically significant differences were observed among the four groups regarding age, hypertension, height, PLT, ALB, serum IgG, or serum IgA levels (*p* > 0.05), as shown in Table 1.

**Table 1.** Anthropometric and clinical characteristics of patients with IgAN categorized by quartile of NHHR.

Clinical indictors	Quartile 1 (*n* = 100)	Quartile 2 (*n* = 100)	Quartile 3 (*n* = 100)	Quartile 4 (*n* = 100)	H/χ²	*P* value
Age (years)	40 (32,54)	43 (33,52)	41 (31,53)	39 (31,53)	1.604	0.659
Male, n (%)	39 (39.0%)	54 (54.0%)	54 (54.0%)	64 (64.0%)	12.789	0.005
Hypertension (n, %)	44 (44.0%)	48 (48.0%)	50 (50.0%)	52 (52.0%)	1.401	0.705
Height (cm)	164.0 (160.0,170.0)	167.5 (160.0,173.0)	165.0 (160.0,172.0)	168.5 (160.0,174.7)	6.786	0.079
Weight (kg)	63.3 (57.8,73.0)	68.0 (60.8,77.9)	70.4 (63.0,79.3)^†^	75.0 (66.6,84.9)^†^	35.912	<0.001
BMI (kg/m^2^)	23.4 (21.5,25.9)	24.9 (21.5,27.5)	25.8 (23.3,28.2)^†^	27.0 (23.9,29.1)^†^	35.054	<0.001
SBP (mmHg)	130 (115,146)	132 (123,147)	137 (121,152)	137 (123,153)^†^	10.490	0.015
DBP (mmHg)	80 (72,90)	84 (77,94)	87 (80,96)^†^	89 (80,98)^†^	16.557	<0.001
Hb (g/L)	130 (121,142)	133 (122,146)	138 (125,150)^†^	140 (126,154)^†^	12.654	0.005
PLT (10^9^/L)	242 (197,273)	233 (202,285)	243 (207,292)	247 (222,293)	3.721	0.293
ALB (g/L)	38.3 (32.9,41.7)	39.2 (34.5,43.3)	40.0 (35.6,43.1)	38.0 (32.7,42.5)	4.598	0.204
TG (mmol/L)	1.12 (0.82,1.59)	1.62 (1.21,2.16)^†^	1.78 (1.39,2.33)^†^	2.47 (1.88,3.55)^†^	113.28	<0.001
TC (mmol/L)	4.10 (3.50,4.84)	4.67 (4.10,5.31)^†^	5.03 (4.36,5.88)^†^	5.32 (4.72,6.35)^†^	66.296	<0.001
HDL-C (mmol/L)	1.39 (1.17,1.62)	1.15 (1.01,1.33)^†^	1.05 (0.94,1.25)^†^	0.95 (0.81,1.13)^†^	94.312	<0.001
LDL-C (mmol/L)	2.26 (1.80,2.78)	2.80 (2.42,3.35)^†^	3.20 (2.56,3.81)^†^	3.30 (2.53,4.19)^†^	66.924	<0.001
BUN (mmol/L)	5.59 (4.65,7.39)	5.83 (4.56,8.13)	6.18 (5.03,8.37)	6.96 (5.23,8.55)^†^	10.773	0.013
Scr (μmol/L)	75.35 (61.87,96.12)	92.30 (68.43,126.4)^†^	89.50 (74.32,124.50)^†^	112.69 (80.90,148.25)^†^	33.246	<0.001
eGFR (mL/min/1.73 m^2^)	96.14 (70.15,113.01)	77.72 (53.63,109.00)	78.50 (53.00,102.64)^†^	67.76 (46.20,89.72)^†^	25.766	<0.001
UA (μmol/L)	360.5 (283.4,414.5)	365.0 (305.8,452.9)	383.7 (337.1,454.3)^†^	400.5 (347.7,460.4)^†^	17.677	<0.001
IgG (g/L)	10.10 (7.58,11.85)	10.40 (8.68,11.87)	10.10 (8.34,12.15)	8.96 (7.36,11.56)	2.422	0.489
IgA (g/L)	3.16 (2.36,3.70)	3.15 (2.50,3.99)	3.26 (2.43,3.97)	3.35 (2.62,3.88)	1.449	0.694
C3 (g/L)	1.05 (0.93,1.23)	1.11 (0.97,1.26)	1.12 (1.00,1.24)	1.20 (1.04,1.38)^†^	21.247	<0.001
C4 (g/L)	0.25 (0.20,0.31)	0.26 (0.21,0.33)	0.27 (0.23,0.34)	0.29 (0.24,0.36)^†^	14.762	0.002
24-h UPE (g/d)	2.16 (1.16,3.82)	2.41 (1.19,4.79)	2.60 (1.45,4.79)	4.16 (1.95,7.93)^†^	18.880	<0.001

BMI: body mass index; SBP: systolic blood pressure; DBP: diastolic blood pressure; Hb: hemoglobin; PLT: platelet count; ALB: albumin; TG: triglyceride; TC: total cholesterol; HDL-C: high-density lipoprotein cholesterol; LDL-C: low-density lipoprotein cholesterol; BUN: blood urea nitrogen; Scr: serum creatinine; eGFR: estimated glomerular filtration rate; UA: uric acid; IgG: serum immunoglobulin G; IgA: serum immunoglobulin A; 24-h UPE: 24-h urine protein electrophoresis.

^†^ *p* < 0.05 compared with Quartile 1.

### 3.2. Associations of NHHR with Oxford classification

**Figure 2.**
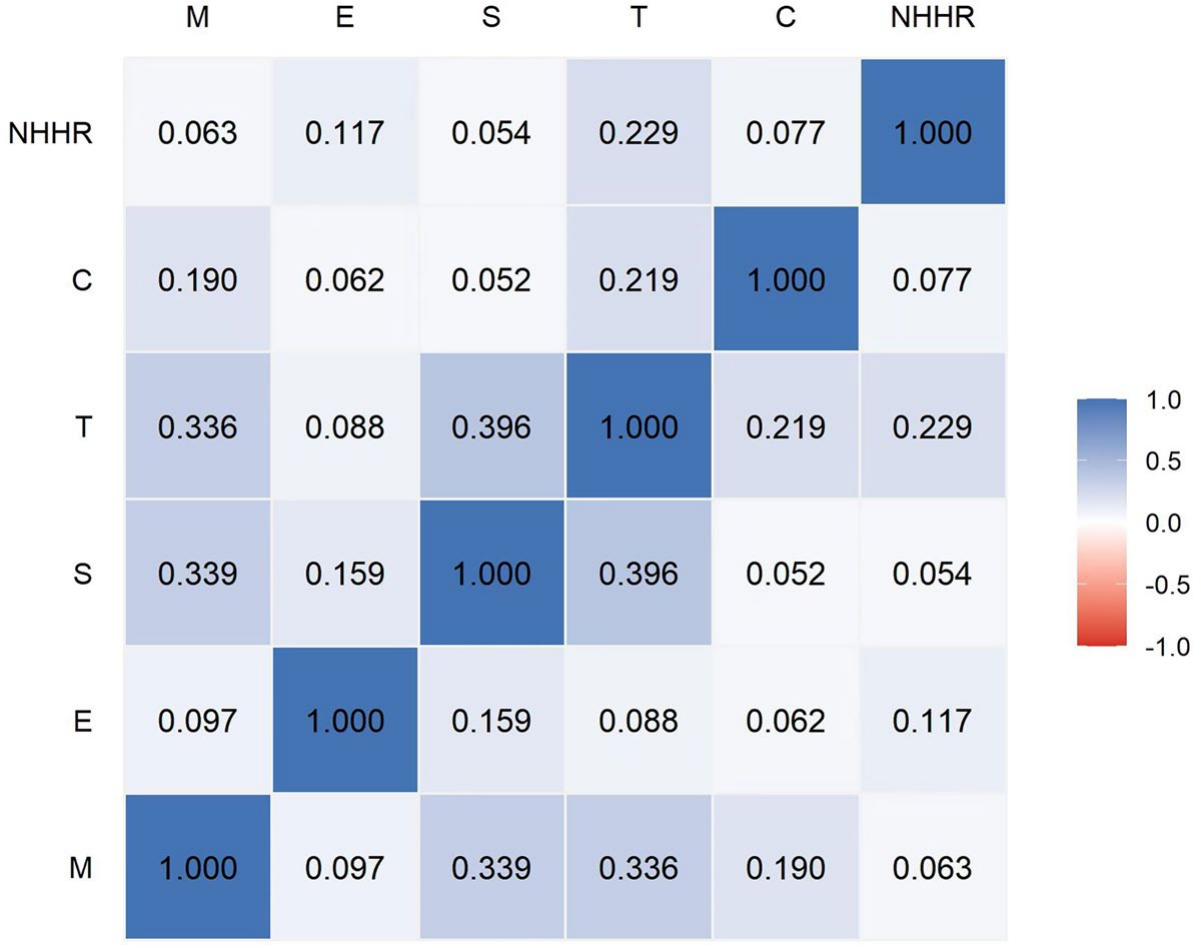
Heat matrix of correlation coefficients between NHHR and Oxford classification in the study population.

Renal pathological findings revealed no statistically significant differences in M, S, or C lesions among the four groups (all *p* > 0.05). In contrast, significant differences were observed in E and T lesions across the groups (all *p* < 0.05). Specifically, compared with the Q1 group, the Q4 group exhibited a higher proportion of E1 and T1/2 lesions (*p* < 0.05), as detailed in Table 2. The correlation between NHHR and histopathological changes in IgAN patients was further evaluated using Spearman’s rank correlation coefficient analysis. The results indicated no correlation between NHHR and M, S, or C lesions (all *p* > 0.05), while significant correlations were found with E and T lesions (all *p* < 0.05). Notably, NHHR showed a positive correlation with T lesions (correlation coefficient = 0.229, *p* < 0.001) (Table 3, Figure 2).

**Table 2.** Comparison of pathological characteristics in patients with IgAN among different NHHR quartile groups.

Pathological characteristics	Score	total	Quartile 1	Quartile 2	Quartile 3	Quartile 4	χ²	*P* value
**Oxford MEST-C, n (%)**								
M	M0	93	26 (26.0%)	25 (25.0%)	23 (23.0%)	19 (19.0%)	1.611	0.657
	M1	307	74 (74.0%)	75 (75.0%)	77 (77.0%)	81 (81.0%)		
E	E0	351	91 (91.0%)	92 (92.0%)	88 (88.0%)	80 (80.0%)	8.256	0.041
	E1	49	9 (9.0%)	8 (8.0%)	12 (12.0%)	20 (20.0%)		
S	S0	231	62 (62.0%)	55 (55.0%)	61 (61.0%)	53 (53.0%)	2.408	0.492
	S1	169	38 (38.0%)	45 (45.0%)	39 (39.0%)	47 (47.0%)		
T	T0	242	76 (76.0%)	62 (62.0%)	61 (61.0%)	43 (43.0%)	22.97	<0.001
	T1/T2	158	24 (24.0%)	38 (38.0%)	39 (39.0%)	57 (57.0%)		
C	C0	313	81 (81.0%)	78 (78.0%)	81 (81.0%)	73 (73.0%)	2.512	0.473
	C1/C2	87	19 (19.0%)	22 (22.0%)	19 (19.0%)	27 (27.0%)		

M: mesangial hypercellularity; E: endocapillary hypercellularity; S: segmental glomerulosclerosis; T: tubular atrophy and interstitial fibrosis; C: crescents.

**Table 3.** Relationship between NHHR and oxford classification in IgAN.

Indexes	Correlation coefficient	95% CI	*P* value
		Lower Upper	
M	0.063	−0.038 to 0.163	0.210
E	0.117	0.016 to 0.215	0.019
S	0.054	−0.047 to 0.154	0.284
T	0.229	0.131 to 0.322	<0.001
C	0.077	−0.024 to 0.177	0.123

M: mesangial hypercellularity; E: endocapillary hypercellularity; S: segmental glomerulosclerosis; T: tubular atrophy and interstitial fibrosis; C: crescents.

### 3.3. Analysis of the influencing factors for T lesions in IgAN

Binary logistic regression analysis was used to identify factors associated with T lesions in IgAN patients, as shown in Table 4. Univariate logistic regression analysis was first performed, and the results showed that NHHR [OR = 1.647, 95% CI (1.337, 2.028), *p* < 0.001], SBP, DBP, Hb, LDL-C,BUN,eGFR,UA,IgG,C4,24h UTP,M1,S1 and C1/C2 were influencing factors for T lesions. Collinearity diagnostics showed that the VIF values of all variables were below 5, indicating no significant multicollinearity among the variables. Subsequently, multivariate logistic regression analysis was performed, incorporating all variables that were significant in univariate analysis (*p* < 0.05). The results showed that elevated NHHR [OR = 1.425, 95% CI (1.046, 1.941), *p* = 0.025] was an independent risk factor for T lesions in IgAN patients.

**Table 4.** Analysis of the influencing factors about T lesions in IgAN.

Variables	Univariate analysis			Multivariate analysis		
	β	OR (95% CI)	*P* Value	β	OR (95% CI)	*P* Value
Age (years)	−0.006	0.994 (0.979–1.009)	0.401			
Male, n (%)	0.323	1.381 (0.922–2.068)	0.117			
BMI (kg/m^2^)	0.004	1.004 (0.951–1.060)	0.891			
SBP (mmHg)	0.027	1.028 (1.017–1.039)	<0.001	0.005	1.005 (0.987–1.024)	0.559
DBP (mmHg)	0.038	1.039 (1.022–1.056)	<0.001	0.028	1.028 (0.997–1.060)	0.073
Hb (g/L)	−0.022	0.978 (0.968–0.988)	<0.001	−0.019	0.981 (0.965–0.996)	0.016
ALB (g/L)	−0.023	0.978 (0.949–1.007)	0.131			
NHHR	0.499	1.647 (1.337–2.028)	<0.001	0.354	1.425 (1.046–1.941)	0.025
TG (mmol/L)	0.064	1.067 (0.892–1.275)	0.479			
TC (mmol/L)	0.118	1.126 (0.985–1.287)	0.083			
HDL-C (mmol/L)	−0.553	0.575 (0.322–1.027)	0.061			
LDL-C (mmol/L)	0.176	1.192 (1.014–1.403)	0.034	0.162	1.176 (0.908–1.523)	0.218
BUN (mmol/L)	0.358	1.431 (1.296–1.580)	<0.001	0.104	1.109 (0.958–1.285)	0.167
eGFR (mL/min/1.73 m^2^)	−0.041	0.959 (0.951–0.968)	<0.001	−0.021	0.979 (0.966–0.993)	0.004
UA (μmol/L)	0.006	1.006 (1.004–1.009)	<0.001	0.004	1.004 (1.000–1.007)	0.026
IgG (g/L)	−0.079	0.924 (0.864–0.988)	0.020	−0.057	0.945 (0.851–1.049)	0.289
IgA (g/L)	−0.142	0.868 (0.725–1.038)	0.121			
C3 (g/L)	−0.354	0.702 (0.302–1.630)	0.410			
C4 (g/L)	2.882	17.842 (2.085–152.670)	0.009	−3.151	0.043 (0.002–1.061)	0.054
24h UTP (g/d)	0.095	1.099 (1.043–1.159)	<0.001	0.073	1.075 (0.993–1.164)	0.072
M1 (vs M0)	2.175	8.802 (4.272–18.136)	<0.001	1.519	4.566 (1.833–11.372)	0.001
E1 (vs E0)	0.535	1.707 (0.937–3.112)	0.081			
S1 (vs S0)	1.707	5.510 (3.561–8.527)	<0.001	1.456	4.287 (2.389–7.695)	<0.001
C1/C2(vs C0)	1.065	2.901 (1.780–4.728)	<0.001	0.836	2.307 (1.151–4.625)	0.018

BMI: body mass index; SBP: systolic blood pressure; DBP: diastolic blood pressure; Hb: hemoglobin; ALB: albumin; NHHR: non-high-density lipoprotein cholesterol to high-density lipoprotein cholesterol ratio; TG: triglyceride; TC: total cholesterol; HDL-C: high-density lipoprotein cholesterol; LDL-C: low -density lipoprotein cholesterol; BUN: blood urea nitrogen; eGFR: estimated glomerular filtration rate; UA: uric acid; IgG: serum immunoglobulin G;IgA: serum immunoglobulin A; 24-h UPE: 24-h urine protein electrophoresis; M: mesangial hypercellularity; E: endocapillary hypercellularity; S: segmental glomerulosclerosis; C: crescents.

### 3.4. Nonlinear relationship between NHHR and T1/T2 lesions in IgAN

**Figure 3.**
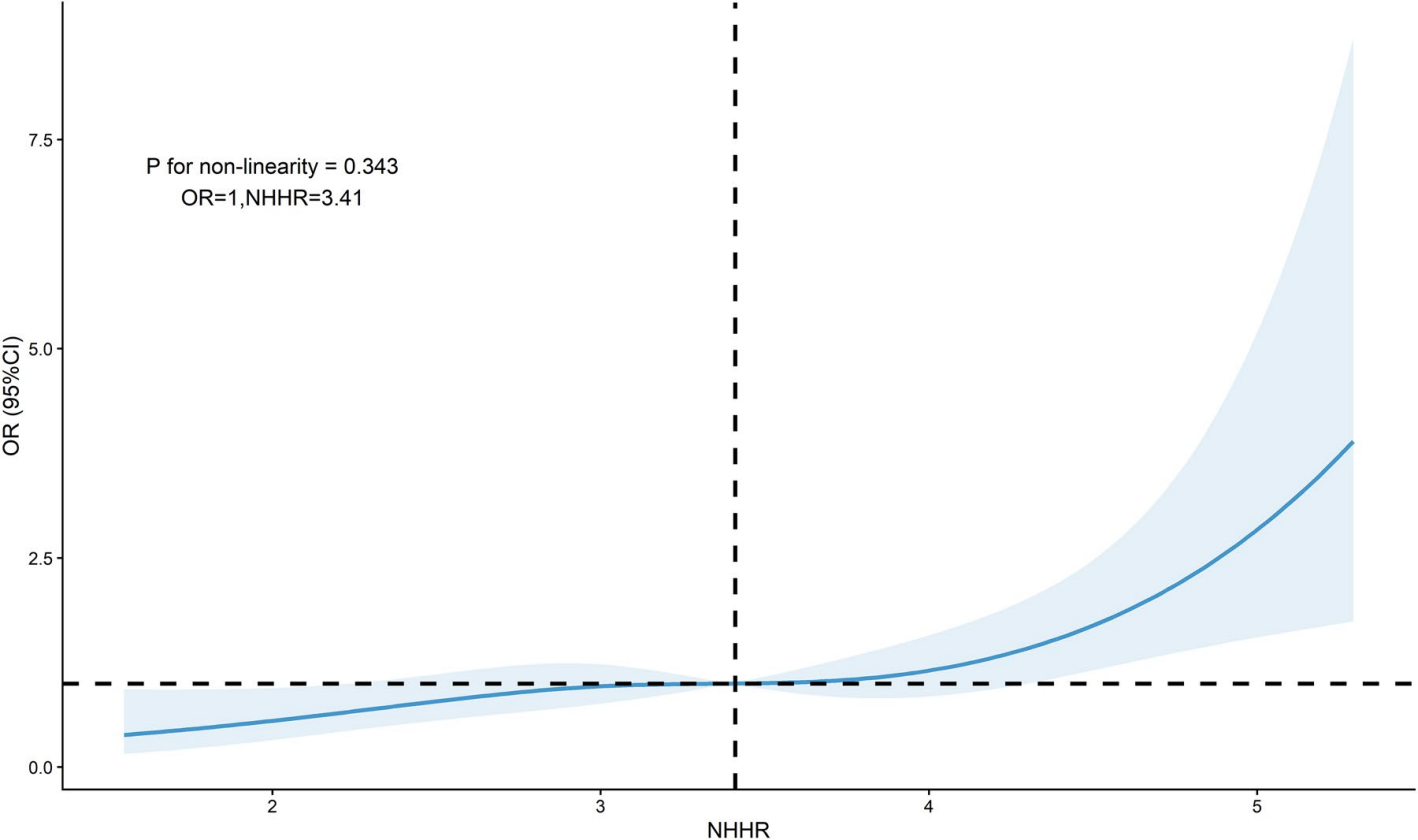
Restricted cubic spline showing the relationship between NHHR and the risk of severe tubular atrophy/interstitial fibrosis.

RCS analysis revealed a linear association between NHHR and T1/T2 lesions in patients with IgAN (*p* for nonlinearity = 0.343). No significant threshold or saturation effect was observed. These results indicate that the risk of developing T1/T2 lesions increases as NHHR increases (Figure 3).

### 3.5. Performance assessment of predictive models for T1/T2 lesions in IgAN

**Figure 4.**
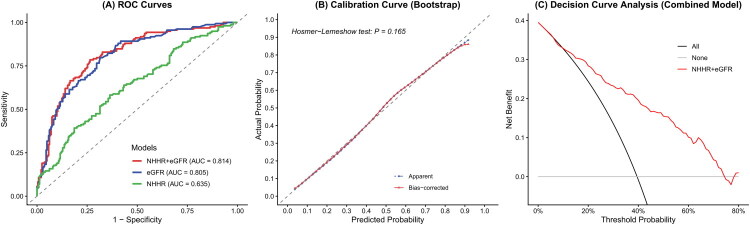
Performance assessment of predictive models for T1/T2 lesions in IgAN. Note: (A) ROC curves for NHHR, eGFR, and the combined model (NHHR+eGFR); (B) Calibration curve of the combined model with 1,000 bootstrap resampling; (C) DCA of the combined model.

ROC curve analysis showed that the area under the curve (AUC) of NHHR for predicting T1/T2 lesions in patients with IgAN was 0.635 (95% CI: 0.580–0.691, *p* < 0.01), with an optimal cutoff value of 3.54, and a sensitivity of 58.2% and a specificity of 63.6%.The combined model (NHHR + eGFR) achieved an AUC of 0.814, slightly higher than that of eGFR alone (0.805). The DeLong test indicated that the difference was statistically significant (*p* < 0.001), although the absolute increment was small (Table S1). The combined model yielded a sensitivity of 78.5% and a specificity of 73.6% (Table 5, Figure 4A). This study performed internal validation using 1,000 bootstrap resampling. The results showed that the combined model had a minimal optimism of only 0.001, and the optimism-corrected AUC remained as high as 0.813 (Table S2). In terms of calibration, the bootstrap internal calibration curve indicated a high degree of consistency between the model-predicted probability of T lesion occurrence and the actual observed probability. Moreover, the Hosmer-Lemeshow test showed no significant bias (*p* = 0.165) (Figure 4B). DCA also demonstrated a net benefit within the threshold probability range of 2%–80% (Figure 4C).

**Table 5.** Predictive value of NHHR, eGFR, and their combination for T1/T2 lesions in patients with IgAN.

	AUC	SE	95% CI
eGFR	0.805	0.022	0.762–0.848
NHHR	0.635	0.028	0.580–0.691
NHHR+eGFR	0.814	0.022	0.771–0.857

### 3.6. Subgroup analysis

**Figure 5.**
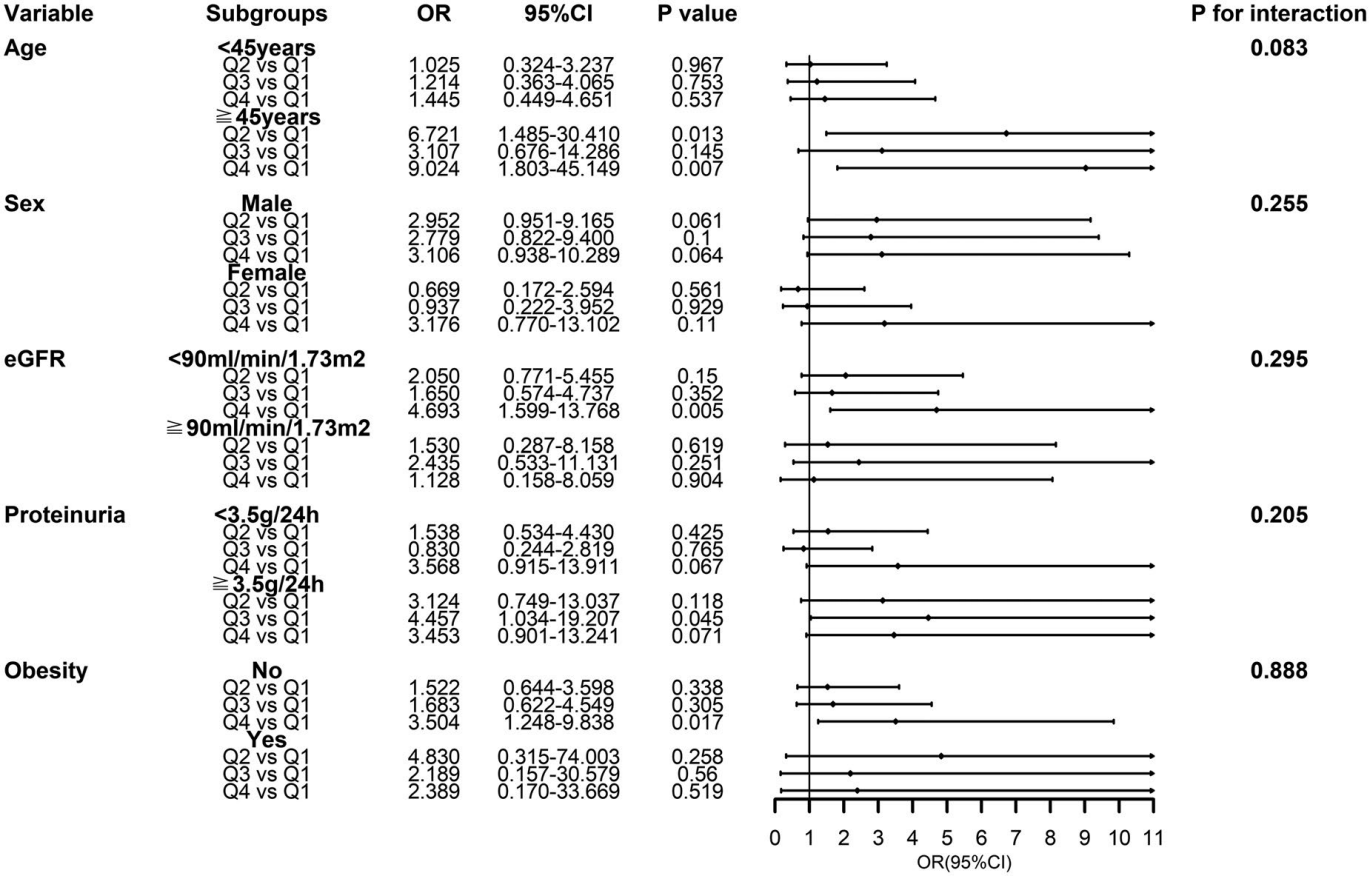
Forest plot showing the risk of T1/T2 lesions in different subgroups of patients with IgAN. All regression models were adjusted for the same set of covariates as the primary multivariate logistic regression analysis in Table 4.

Subgroup analysis was performed to assess whether the association between NHHR and T1/T2 lesions varied across different clinical subgroups of patients with IgAN. All subgroup analyses adopted the same multivariate adjustment model as the primary analysis (Table 4), to control for potential confounding factors. No statistically significant interaction effects were found between NHHR and any subgroup variables (all *p* for interaction > 0.05) (Figure 5).

## 4. Discussion

IgAN is one of the leading causes of CKD. In addition to the characteristic deposition of IgA in the glomerular mesangium, its pathological features often include tubulointerstitial lesions (TIL). Notably, the severity of TIL is a more accurate predictor of renal function progression than glomerular pathology, making it a major focus of clinical attention [19]^.^ With lifestyle changes, the prevalence of dyslipidemia has increased markedly. Recent studies have shown a close association between dyslipidemia and pathological injury in IgAN [3]. Dyslipidemia is defined as elevated levels of TG, LDL-C, or TC, or a reduced level of HDL-C. Recently, NHHR—a novel lipid ratio derived from the combination of non-HDL-C and HDL-C—has emerged as a relatively low-cost and easily obtainable indicator. Studies have confirmed that NHHR is closely associated with kidney diseases [7]. Although the relationship between NHHR and T lesions in patients with IgAN has not been clearly established, previous studies have shown that patients with low HDL-C are more likely to develop severe tubular atrophy/interstitial fibrosis [17]. This provides indirect evidence for the potential value of NHHR in assessing tubular atrophy/interstitial fibrosis in patients with IgAN.

In this study, a cross-sectional analysis was performed on 400 patients with IgAN to investigate the potential correlation between NHHR and T lesions. The results showed that patients with higher NHHR had a significantly higher incidence of T1/T2 lesions. Multivariate logistic regression analysis confirmed that an elevated NHHR is an independent risk factor for T1/T2 lesions in IgAN patients after adjusting for confounding factors including eGFR and proteinuria. RCS analysis demonstrated a linear positive correlation between NHHR and the risk of T1/T2 lesions, indicating that the risk increases with rising NHHR.ROC curve analysis identified an optimal NHHR cutoff of 3.54, although the predictive accuracy of NHHR alone was modest (AUC = 0.635). Given the limited discriminatory ability, this cutoff should be interpreted with caution and requires external validation. The addition of NHHR to eGFR provided a statistically significant but numerically small improvement in AUC, suggesting that NHHR may serve more appropriately as an adjunctive marker to eGFR rather than a substitute. Whether NHHR levels above 3.54 have clinical utility in guiding monitoring strategies remains to be determined in future studies. Subgroup analysis stratified by age, sex, eGFR, proteinuria levels, and obesity status showed that the association between NHHR and the risk of T1/T2 lesions was consistent across different clinical subgroups. Although interaction terms were not statistically significant (all *P* for interaction > 0.05). The association was more pronounced in patients who were older (≥45 years), had lower renal function (eGFR < 90 mL/min/1.73m^2^), had heavy proteinuria (≥3.5 g/24 h),or were non-obese.

The exact mechanism by which elevated NHHR promotes the development of T lesions in patients with IgAN remains unclear, but several possible explanations exist. First, an elevated NHHR is usually characterized by high levels of non-HDL-C (such as LDL-C) and low levels of HDL-C [20]. Under normal conditions, renal cells take up LDL through LDL receptors and break it down into free cholesterol (FC) and unsaturated fatty acids. This process is strictly regulated by intracellular FC levels; when intracellular FC levels rise, the expression of LDL receptors on the cell surface is downregulated. However, under oxidative stress, LDL deposition and oxidation in the vascular endothelium accelerate. Once oxidized to oxidized LDL (ox-LDL), it is taken up by cells through scavenger receptors (SRs), which are not regulated by intracellular FC feedback. Hypercholesterolemia can further upregulate SR expression. This process leads to monocyte/macrophage infiltration and the formation of foam cells in lesion areas after the uptake of ox-LDL [21,22]. Accumulated ox-LDL can directly induce epithelial-mesenchymal transition (EMT) in renal tubular epithelial cells, serving as a key initiating factor in renal interstitial fibrosis. *In vitro* studies have confirmed that ox-LDL downregulates the expression of the tubular epithelial marker E-cadherin and upregulates the level of the mesenchymal marker α-SMA, causing renal tubular epithelial cells to lose their inherent morphology and function while acquiring a mesenchymal proliferative and migratory phenotype. Simultaneously, ox-LDL promotes the excessive synthesis of extracellular matrix components such as type I collagen and fibronectin, leading to abnormal renal matrix deposition [23,24]. Furthermore, impaired cholesterol efflux mediated by L-HDL exacerbates lipid accumulation in renal tubular epithelial cells, induces endoplasmic reticulum stress and mitochondrial dysfunction, aggravates tubular cell injury, and thereby drives the fibrotic process [25,26]. Abnormal lipid accumulation can also activate a vicious cycle of oxidative stress and inflammation, continuously aggravating kidney injury. Elevated non-HDL-C activates NADPH oxidase, inducing the generation of large amounts of reactive oxygen species (ROS). ROS not only directly damage renal tubular structures but also activate the NF-κB pathway, promoting the expression of inflammatory factors such as MCP-1 and IL-1β, thereby amplifying the local inflammatory response in the kidney [27]. Meanwhile, foam cells can produce various pro-inflammatory cytokines, chemokines, and growth factors. Among these, IL-6 can upregulate the expression of TGF-β1 and enhance the activity of the TGF-β1-Smad signaling pathway [28]. It also promotes the expression of cell adhesion molecules, induces inflammatory cell recruitment, and stimulates fibroblast proliferation and collagen synthesis. At the same time, TNF-α can promote fibronectin and proteoglycan synthesis by renal tubular epithelial cells, accelerate fibroblast formation, and lead to tubulointerstitial damage [29].

Second, previous studies have found that patients with L-HDL have more severe T lesions than those without L-HDL [30]. HDL is a key regulator of cholesterol metabolism and plays a central role in maintaining systemic lipid homeostasis by mediating reverse cholesterol transport from peripheral tissues, including the kidneys, to the liver [30]. Under pathological conditions, HDL undergoes both a quantitative reduction and functional impairment. This leads to a loss of its antioxidant and anti-inflammatory properties, increased transformation of monocytes/macrophages into foam cells, and the initiation of pro-inflammatory cascades. This creates a vicious cycle with oxidative stress and chronic inflammation, resulting in endothelial dysfunction, which accelerates atherosclerosis and aggravates kidney injury [31]. Third, dyslipidemia can cause lipoprotein deposition in the intima of renal vessel walls. The proliferation of vascular smooth muscle cells promotes plaque formation, which leads to renal arteriosclerosis, ischemic-hypoxic kidney injury, and fibrosis of intrinsic renal cells, ultimately resulting in progressive renal interstitial fibrosis [32].

In this study, multivariate logistic regression analysis showed that, in addition to traditional risk factors such as eGFR, the occurrence of T1/T2 lesions in patients with IgAN was also significantly associated with decreased Hb, elevated UA, and specific renal pathological features (M1, S1, C1/C2).

Existing evidence indicates that patients with IgAN who have renal anemia show a significantly higher incidence of T1/T2 lesions than those without anemia. Multivariate regression analysis has also confirmed an independent association between renal anemia and T1/T2 lesions, with the degree of anemia being more severe in patients with T2 lesions than in those with T0 or T1 lesions [33]. This is consistent with our findings and suggests that the presence of renal anemia in patients with IgAN should raise suspicion for concomitant tubulointerstitial lesions. The underlying mechanism may be primarily related to impaired erythropoietin (EPO) production [34]. Furthermore, several recent studies have shown that patients with IgAN who have hyperuricemia often exhibit more severe renal tubular atrophy/interstitial fibrosis [35,36]. This may be related to the conversion of UA into urate crystals, which deposit in the renal tubules and interstitium, trigger inflammatory responses and fibrosis, and thereby directly contribute to renal pathological injury [37].

Previous studies have reported that cytokines produced by mesangial cell proliferation in IgAN can act on podocytes, inducing podocyte apoptosis and aggravating tubulointerstitial injury [38]. In this study, multivariate logistic regression analysis showed that M1 lesions were an independent risk factor for the development of T1/T2 lesions. We also observed that patients with IgAN who had S1 lesions often showed more severe tubular atrophy/interstitial fibrosis. Research suggests that deposition of plasma IgA in the glomerular mesangium can activate podocyte injury pathways, leading to podocyte adhesion and subsequent interstitial fibrosis through podocyte–tubulointerstitial crosstalk and epithelial cell migration [39]. Additionally, the presence of C1/C2 lesions was associated with an increased risk of tubular atrophy/interstitial fibrosis. Studies have shown that scores for interstitial inflammatory cell infiltration, interstitial fibrosis, and tubular atrophy are significantly higher in patients with crescent formation than in those without. These findings suggest that patients with crescent formation have more severe tubulointerstitial injury and a poorer prognosis [40].

Subgroup analysis revealed that the association between NHHR and T1/T2 lesions was stronger in older patients with IgAN. This may be related to age-associated structural and functional changes in the kidneys. With advancing age, renal blood flow decreases, renal vessels become less compliant, systemic and local renal inflammatory responses increase, and the kidney’s compensatory capacity declines [41]. In addition, the present study found that the association between NHHR and T1/T2 lesions was more pronounced in non-obese IgAN patients. Obesity itself exhibits a certain degree of pro-inflammatory and pro-fibrotic characteristics, manifested as adipocyte hypertrophy, dysregulated secretion of adipokines (e.g. leptin), and systemic low-grade inflammation [42]. These pathological pathways may participate in promoting tubular atrophy and interstitial fibrosis, rendering obesity a potential contributing factor to T lesions. In this context, obesity-related metabolic signals may, to some extent, interfere with or attenuate the independent association between NHHR and T lesions. In contrast, such interfering factors are relatively weaker in non-obese patients, and consequently, the association between NHHR and T lesions may be more readily detected. The present study also found that the association between NHHR and T lesions may be influenced by renal function level. Subgroup analysis further revealed that the association between NHHR and T lesions was more pronounced in patients with an eGFR < 90 mL/min/1.73m^2^. A plausible explanation is that NHHR, as a composite lipid metric, reflects the overall homeostasis of lipid metabolism rather than a single measure of nutritional status. When renal function declines to a certain threshold (e.g. eGFR < 90mL/min/1.73m^2^),the renal capacity for lipoprotein metabolism and clearance may start to decrease, thereby potentially making the lipid metabolic balance more prone to disruption. In this context, an elevated NHHR may serve as a more sensitive indicator of metabolic dysregulation, thereby demonstrating a stronger association with T lesions. It should be noted that, given the limited sample size and the exploratory nature of subgroup analyses in this study, the above findings warrant further validation in larger cohort studies.

This study has several strengths. It is the first to investigate the relationship between NHHR levels and T lesions in patients with IgAN. In addition, subgroup analyses and interaction tests were performed to explore potential differences across subgroups. However, this study also has some limitations. As a single-center retrospective cross-sectional study, causal inference between NHHR and T lesions in IgAN patients is not possible, and potential confounders (e.g. dietary habits, genetic background, smoking status) were not fully excluded. No prognostic follow-up was done, so NHHR’s value for predicting long-term renal outcomes could not be assessed. Owing to study constraints, no immunohistochemical staining for lipid-related and inflammatory markers (e.g. ox-LDL, CD68, IL-6/TGF-β1) was performed on the renal biopsy specimens, precluding direct verification of the causal mechanism of NHHR in T lesions. Furthermore, theoretically, T lesions themselves may influence lipid profiles by affecting the activity of lipid metabolism-related enzymes or altering the reabsorption function of lipoproteins in renal tubules, thereby potentially impacting lipid ratios such as NHHR and TG/HDL. Thus, a bidirectional relationship between NHHR and T lesions may exist. Additionally, the optimal NHHR cut-off of 3.54 (single-center data) awaits external validation. For future research, large-scale prospective multicenter cohort studies with long-term follow-up are needed to validate NHHR’s predictive value for T lesion progression. Lipidomic analyses, *in vitro*/*in vivo* experiments and matched biomarker detection should clarify the underlying molecular mechanisms. If feasible, molecular pathological staining of renal biopsy specimens will advance this associative study to mechanistic exploration.

## 5. Conclusion

In conclusion, an elevated NHHR is independently associated with T lesions in this single-center, cross-sectional cohort of patients with IgAN. While a cut-off of 3.54 was identified in the present study, this remains to be validated in external cohorts.

## Supplementary Material

Supplemental Material

## Data Availability

The datasets used and/or analyzed during the current study are available from the corresponding author on reasonable request.
