# Supplementary material for: The relationship between the non–high-density lipoprotein cholesterol to high-density lipoprotein cholesterol ratio and tubular atrophy/interstitial fibrosis in patients with IgA nephropathy
Source: Ren Fail. 2026 Jul 14;48(1):2699010. doi: 10.1080/0886022X.2026.2699010 (PMC13374780; doi:10.1080/0886022X.2026.2699010)
Supplement: Supplemental Material [file IRNF_A_2699010_SM6639.docx]

Supplementary Table S1 Comparison of AUCs between NHHR+eGFR and eGFR using DeLong’s test

| **Test pair** | **z** | ***P* value** | **Difference in AUC** | **Standard error of difference** | **95% CI** |
| --- | --- | --- | --- | --- | --- |
| NHHR + eGFR vs eGFR | 14.156 | <0.001 | 0.619 | 0.212 | 0.533-0.704 |

Supplementary Table S2 Apparent AUC, optimism, and corrected AUC of each prediction model

| **Models** | **Apparent_AUC** | **Optimism** | **Corrected_AUC** |
| --- | --- | --- | --- |
| NHHR | 0.635 | -0.000 | 0.635 |
| eGFR | 0.805 | -0.001 | 0.806 |
| Combined (NHHR + eGFR) | 0.814 | 0.001 | 0.813 |
